# Evaluation of dry eye disease in newly diagnosed anxiety and depression patients using anterior segment optical coherence tomography

**DOI:** 10.1186/s40662-019-0149-y

**Published:** 2019-08-09

**Authors:** Mahmut Oğuz Ulusoy, Selen Işık-Ulusoy, Sertaç Argun Kıvanç

**Affiliations:** 1 Department of Ophthalmology, Başkent University School of Medicine Konya Research Hospital, 42000 Konya, Turkey; 2 Department of Psychiatry, Başkent University School of Medicine Konya Research Hospital, Konya, Turkey; 3 Department of Ophthalmology, Uludağ University School of Medicine, Bursa, Turkey

**Keywords:** Depression, Anxiety, Dry eye, Tear meniscus, Anterior segment optical coherence tomography

## Abstract

**Background:**

We aimed to evaluate dry eye diseases (DED) in patients with newly diagnosed depression and anxiety patients.

**Methods:**

Forty newly diagnosed depression, 35 anxiety patients, and 37 controls without any history of taking psychiatric drugs (or before the beginning of psychiatric medication) and topical ophthalmic drop use, were included in the study. All depression and anxiety diagnoses were performed by an experienced psychiatrist. Beck depression and anxiety tests were used to measure disease severity. Tear film break up time (TBUT), Schirmer’s test, Oxford scores and ocular surface disease index (OSDI) were admiinistered to participants. Anterior segment optical coherence tomography was used to measure tear meniscus heights (TMH), tear meniscus depths (TMD) and tear meniscus areas (TMA).

**Results:**

In anxiety and depression groups, Schirmer’s test (mm) (7.24 ± 6.02, 6.58 ± 4.9 and 18.79 ± 4.9 respectively, *p* < 0.05) and TBUT (s) (5.62 ± 3.1, 5.6 ± 3.5 and 13.37 ± 1.7 respectively, *p* < 0.05) were significantly lower than control group. In addition, OSDI and Oxford scores were significantly higher than controls. OSDI scores were 28.01 ± 19, 30.43 ± 18.49, 14.38 ± 8.14 respectively (*p* = 0.002) and Oxford scores were 1.9 ± 0.7, 2.1 ± 0.6 and 0.7 ± 0.4 respectively (*p* = 0.001). TMD, TMH and TMA values were significantly lower in anxiety and depression groups compared with control groups. Correlations between disease inventory scores and dry eye tests were detected.

**Conclusions:**

This study showed a relation between DED and newly diagnosed anxiety and depression patients with no history of psychiatric drug use. The presence of correlation between dry eye tests and disease inventory scores strengthens this association. This is an important knowledge that need to be evaluated in these patients before starting psychiatric medication.

## Background

Dry eye disease (DED) is a common disease that affects the ocular surface with reported prevalence rates changes from 11 to 54%. [1] It is a multifactorial disease of the tear film and ocular surface that results in symptoms of discomfort such as pain, heaviness, grittiness, burning, dryness, itchiness, foreign body sensation, visual disturbances, and tear film instability according to the International Dry Eye Workshop. [2] Depression is associated with comorbidities such as hypothyroidism, systemic lupus erythematosus, myasthenia gravis, liver diseases, hypertension, diabetes mellitus, cardiac disease, gastrointestinal disorders, rheumatic diseases, and systemic medications. [1, 3, 4] In addition, an association between DED and depression or anxiety treatment was reported in several studies. [5–7] The prevalence of DED symptoms in depressive and anxiety patients ranged from 21 to 52% in previous studies. [8–10] However, the main cause of DED in those patients is uncertain, whether due to illness, medication or both.

The aim of this study was to evaluate the ocular surface and anterior segment optical coherence tomography (AS-OCT) findings in patients who had depression and anxiety disorders, but without any history of psychiatric drug use.

## Methods

### Design of Study Groups

This study was conducted at Başkent University School of Medicine, Konya Research Hospital, Department of Ophthalmology, and approved by the Institutional Review Board and Ethics Committee of the same University (Project No: KA17/106). This study adhered to the tenets of the Declaration of Helsinki and informed consent was obtained from all patients. Forty newly diagnosed depressive disorder patients, 35 newly diagnosed anxiety disorder patient and 37 healthy control patients were recruited in this study. Newly diagnosed anxiety and depression patients were referred to the ophthalmology department immediately after diagnois before initiation of psychiatric medication. Patients without any history of dry eye, and systemic and topical drug use, were selected for the study.

### Psychiatric diagnoses

The Structured Clinical Interview for Diagnostic and Statistical Manual of Mental Disorders-IV (DSM-IV) Axis I disorders (SCID-I) was used for psychiatric disorders. [11] The interview was done by an experienced psychiatrist that was unaware of ocular findings and scores on all self-reported questionnaires (Beck Depression Index (BDI) and Beck Anxiety Inventory (BAI)).

BDI and BAI were used for evaluating patient mental health. The questionnaire consists of 21 items that scale, from 0 (neutral) to 3 (maximum intensity), and the final score range was from 0 to 63 points.

For BAI scores were divided into four degrees due to disease severity. (0 to 7 indicates normal range, 8–15 mild anxiety, 16–25 moderate anxiety and 26–63 severe anxiety). [12] Overall reliability of the scales which was adapted to Turkish version computed by Cronbach’s alfa was 0.90.

For BDI scores of 0–9 are normal, scores of 10–16 indicate mild depression, scores of 17–29 indicate moderate depression and scores of 26–63 indicate severe symptoms of depression. [13]

Psychiatric exclusion criteria from the study were previous medication for anxiety or depressive disorder, a history of seizure disorder, serious or unstable medical illness, substance abuse disorder (active within the past year), serious suicidal risk, schizophrenia, or major antisocial personality disorder.

After psychiatric diseases were diagnosed, patients were referred to the Ophthalmology Department.

### Ophthalmic examinations

Detailed full ophthalmic examination was done for all patients such as visual acuity (by Snellen chart), refraction assessment, biomicroscopic examination, intraocular pressure measurement and fundus examination.

Patients completed the ocular surface disease index (OSDI) at initial visit. [8] AS-OCT tear meniscus parameters, Schirmer, tear break-up time (TBUT) and corneal staining scores (Oxford Scheme) were measured for all patients. [14]

**Fig. 1:**
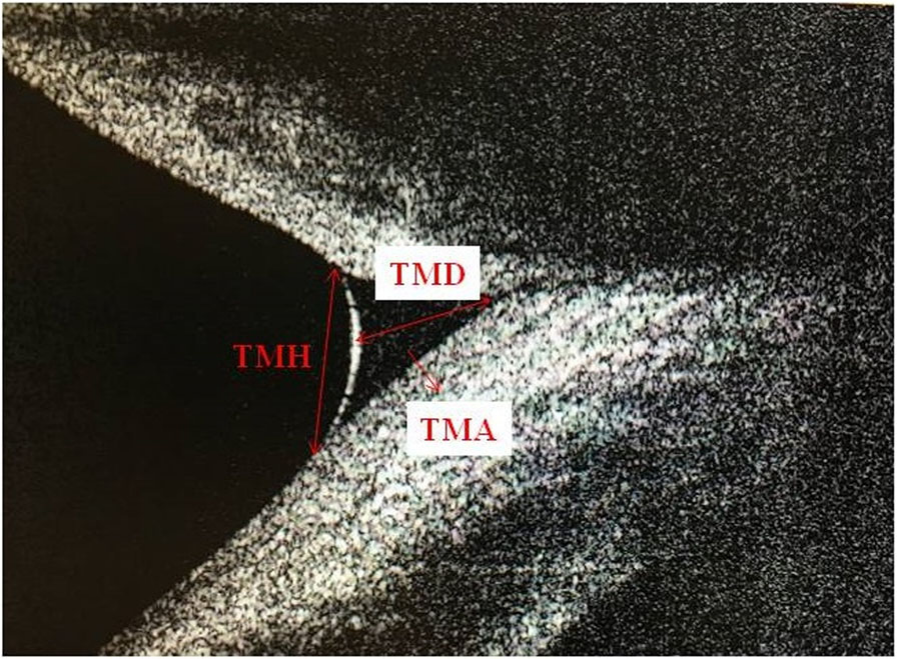
Image of measurement of tear meniscus height, tear meniscus depth and tear meniscus area using anterior segment- optical coherence tomography

A spectral domain optical coherence system (RTVue-100; Optovue, Fremont, CA) with a corneal adaptor module was used. This system has a 6-mm vertical beam that takes 26,000 axial scans per second and has a 5-mm axial resolution to a depth of 2.8 mm. Vertical images were recorded at the 6-o’clock position of the cornea 3 s after each blink, which was repeated 3 times, and a built-in calliper was used to measure tear meniscus heights (TMH), tear meniscus depths (TMD) and tear meniscus areas (TMA). The mean of the 3 measurements was used for analysis. TMH was determined as the length from the point where the meniscus intersected with the cornea superiorly to the eyelid inferiorly. TMD was determined as the length from the apex of the fornix to the surface of the tear meniscus, perpendicular to TMH. The borders of the tear meniscus were marked with a calliper, and integrated analysis software calculated the area in mm^2^ to measure TMA. Only measurements of the right eye were used for statistical analysis. (Fig. 1).

Corneal staining scores were classified as 0 to 5 based on the Oxford Scheme. [14] Finally, Schirmer test was performed for a duration of 5 min without topical anaesthetic drops. The filter paper strip was placed in the middle and lateral thirds of the lower eyelid.

### Dry eye diagnosis

Schirmer’s test was used to quantify the tear secretion within 5 min by wetting of the filter paper placed on the ocular surface. Schirmer < 10 mm in the unanaesthetized eye is considered to be consistent with a disturbance in the tear film. TBUT was measured after impregnated of a moistened fluorescein 1-mg strip in the lateral one-third of the patient’s lower eyelid. The interval between the last complete blink and the appearance of the first corneal black spot in the stained tear film was measured. TBUT is accepted as evidence for dry eye, if it is shorter than 10 s. The Oxford scoring system is as follows with 1 = mild, 2 = moderate, and scores of > 3 being consistent with severe DED.

Patients with a history of ocular surface diseases and inflammation, contact lens wearing, any ocular surgery, and any systemic disease that could affect measurements were excluded. Also use of any eye medications or artificial tears during the previous month were excluded.

#### Statistical analysis

SPSS version 21.0 (SPSS, Chicago, IL, USA) was used for statistical analysis. The normality of the values was analysed using the Kolmogorov-Smirnov test. One-way ANOVA test followed by Tukey’s post hoc was used to analyse the difference between the three groups. Correlations between the variables were investigated using the Pearson or Spearman correlation coefficient. *p* < 0.05 was considered as significant.

## Results

The average age in years did not differ significantly between the three groups (38.8 ± 10.6 in the depression group, 40.7 ± 14.8 in the anxiety group and 39.4 ± 6.1 in the control group, *p* = 0.765). Male-to-female ratio is 11/29 in the depression group, 9/26 in the anxiety group and 10/27 in control group (*p* = 0.658). Mean BDI of depression and control groups were 24.25 ± 6.7 and 11.24 ± 8.2, respectively, (*p* = 0.001). Mean BAI of anxiety and control groups were 23.59 ± 9.1 and 13.51 ± 9.6, respectively, (*p* = 0.002).

Conventional dry eye tests were significantly different in depression and anxiety groups compared with the control group. As expected Schirmer test and TBUT were lower and OSDI and corneal staining scores were higher in the patient groups than in controls. (*p* < 0.05 for Schirmer test, TBUT, OSDI and corneal staining scores). Tear meniscus parameters for the depression and anxiety groups were significantly lower compared with the control group (*p* < 0.05 for TMH, TMD, and TMA). Demographic characteristics, Schirmer, TBUT, OSDI, staining scores, tear meniscus parameters and psychiatric test scores are listed in Tables 1 and 2.

**Table 1:** Demographic factors, disease severity index and conventional dry eye test results for all groups

	Depression	Anxiety	Control	*p*		
				Dep vs. Anx	Dep vs. Control	Anx vs. Control
Age (years)	38.8 ± 10.6	40.7 ± 14.8	39.4 ± 6.1	0.74	0.86	0.78
Gender (M/F)	11/29	9/26	10/27	0.65	0.71	0.73
Schirmer’s test (mm)	6.58 ± 4.9	7.24 ± 6.02	18.79 ± 4.9	0.16	< 0.001	< 0.001
TBUT (s)	5.6 ± 3.5	5.62 ± 3.1	13.37 ± 1.7	0.45	< 0.001	< 0.001
OSDI	30.43 ± 18.49	28.01 ± 19	14.38 ± 8.14	0.32	0.002	0.012
Oxford score	2.1 ± 0.6	1.9 ± 0.7	0.7 ± 0.4	0.29	< 0.001	< 0.001
				*p*		
BDI	24.25 ± 6.7		11.24 ± 8.2	0.001		
BAI		23.59 ± 9.1	13.51 ± 9.6	0.002		

*M*= male, *F*= female, *BDI*= Beck depression inventory, *BAI*= Beck anxiety inventory, *TBUT*= tear break up time, *OSDI*= ocular surface disease index

**Table 2:** Comparison of AS-OCT parameters among depression, anxiety and control groups

	Depression	Anxiety	Control	*p*		
				Dep vs. Anx	Dep vs. Control	Anx vs. Control
TMH (μm)	215.28 ± 55.7	198.82 ± 48.09	465.29 ± 183.25	0.81	< 0.001	< 0.001
TMD (μm)	149.1 ± 35.41	125.68 ± 29.72	260.97 ± 91.06	0.21	< 0.001	< 0.001
TMA (mm^2^)	0.01 ± 0.005	0.01 ± 0.004	0.11 ± 0.02	0.97	< 0.001	< 0.001

*AS-OCT*= anterior segment optical coherence tomography, *TMH*= tear meniscus height, *TMD*= tear meniscus depth, *TMA*= tear meniscus area

In the depression group, Schirmer’s test was significantly correlated with all tear meniscus parameters; TBUT was correlated with TMH and TMA, OSDI and corneal staining scores were correlated with only TMA (Table 3). However, in the anxiety group, while Schirmer test was significantly correlated with all tear meniscus parameters, TBUT was correlated with TMD and TMA, OSDI was correlated with TMH and TMA parameters (Table 4).

**Table 3:** Correlation between AS-OCT parameters and conventional dry eye tests in patients with depression

Conventional Tests	AS-OCT PARAMETERS					
	TMH		TMD		TMA	
	r	p	r	p	r	P
Schirmer’s test (mm)	0.479	0.01	0.405	0.002	0.548	< 0.001
TBUT (s)	0.405	0.012	0.287	0.81	0.392	0.015
Oxford Score	−0.189	0.35	−0.361	0.26	−0.425	0.032
OSDI	−0.256	0.11	−0.254	0.11	−0.318	0.045

*AS-OCT*= anterior segment optical coherence tomography, *TBUT*= tear break up time, *OSDI*= ocular surface disease index, *TMH*= tear meniscus height, *TMD*= tear meniscus depth, *TMA*= tear meniscus area

**Table 4:** Correlation between AS-OCT parameters and conventional dry eye tests in patients with anxiety

Conventional Tests	AS-OCT PARAMETERS					
	TMH		TMD		TMA	
	r	p	r	p	r	P
Schirmer’s test (mm)	0.622	0.02	0.538	0.03	0.778	0.01
TBUT (s)	0.182	0.3	0.674	0.01	0.682	0.03
Oxford Score	−0.219	0.27	−0.251	0.45	−0.487	0.32
OSDI	−0.646	< 0.05	−0.184	0.64	−0.533	0.018

*AS-OCT*= anterior segment optical coherence tomography, *TBUT*= tear break up time, *OSDI*= ocular surface disease index, *TMH*= tear meniscus height, *TMD*= tear meniscus depth, *TMA*= tear meniscus area

BAI score was correlated with TBUT (r = − 0.423, *p* = 0.013) in patients with anxiety, and BDI was correlated with TBUT (r = − 0.343, *p* = 0.035), TMD (r = − 0.45, *p* = 0.004), TMH (r = − 0.448, *p* = 0.004) and TMA (r = − 0.454, *p* = 0.003) in patients with depression.

## Discussion

Depression and anxiety are associated with DED, however, there is not enough evidence that have evaluated the effects of anxiety and depression to DED. [5] Tıskaoğlu et al. reported that newly diagnosed depressive disorder patients were associated with DED. [15] They suggested that disturbances of the serotonin receptors, which are located around the conjunctival epithelium, can influence meibomian glands, and this can lead to tear film deficiency in depression patients. [16] Consistent with this hypothesis, a recent study reported that patients with DED have higher levels of serotonin in their tears compared with individuals without DED. [17] In the same study, patients with DED symptoms with normal tear production had similar serotonin levels with patients without DED symptoms. [17] These patients correspond to patients with chronic ocular pain TBUT without any defect on tear film parameters. [17] The authors have explained this situation as these patients may have developed central sensitization with DED-associated neuropathic pain e.g. chronic pain symptoms. [17] Others suggested that chronic pain is a potent stress factor that affects mood and have association with mood disorders like depression and anxiety. [18] Chronic pain due to DED, may cause or aggravate the symptoms of these mood disorders. In addition, depression and pain use same biological pathways and neurotransmitters such as adrenaline and serotonin. [19, 20] According to these information, a study became noteworthy which showed the decreased platelet serotonin levels in patients with primary Sjögren’s syndrome akin to depression patients. [21] Any disturbance in meibomian glands may lead to irregularity in secretion of the lipid layer and thus may explain the correlation of short TBUT with depression and anxiety scores. Therefore, these results suggest that, there can be a pathophysiologic intersection between DED and mood disorders.

Our study showed an association between DED and newly diagnosed depression and anxiety. Schirmer’s test, TBUT, OSDI and corneal staining scores were significantly different in the depression and anxiety groups compared with control group. In a similar report, Tıskaoğlu et al. showed an association between DED and newly diagnosed depressive disorder. [15] However, in this study, our patients’ Schirmer and TBUT results were lower while, OSDI scores and corneal staining scores were similar.

One strongest part of this study was, our study was designed with newly diagnosed patients without any systemic psychiatric drug (before beginning of psychiatric medication) and/or ocular drop usage. Previous studies, which reported association between DED and depression and anxiety, evaluated patients that already use antidepressant and antianxiety treatment. [1, 22–24] Therefore, it became very different to discriminate whether DED was caused from medical treatment or illness itself. Results from a previous study, showed a correlation between DED and duration of psychiatric disease, selective serotonin reuptake inhibitors (SSRIs) use and duration of SSRIs use, which supports that these reports did not find out the own effect of diseases. [1] Furthermore, we evaluated newly diagnosed anxiety patients in our study. Similarly, anxiety patients’ dry eye parameters were significantly different from control group. Another novel part of this study was usage of anterior segment OCT in depression and anxiety patients.

We also evaluated using AS-OCT tear meniscus parameters of newly diagnosed depressive and anxiety disorder patients. The AS-OCT is a non-invasive method with detailed images, and it makes it possible to see the meniscus clearly. Tear meniscus change can be evaluated with TBUT test easily. [25–27] All tear meniscus parameters, including TMA, TMH and TMD, of the depression and anxiety groups were significantly lower compared with the control group. As far as we know, there was no study that had evaluated the DED in mood disorders with AS-OCT. Although, conventional DED diagnose tests are minimally invasive, anxiety of the test during application may affect the results, especially in anxiety patients. Therefore, the non-invasive nature of this method became important for these patients.

Similar to previous studies we also examined the correlation between DED tests and BDI and BAI. [15, 23, 24, 28] Previous studies did not report a correlation between these parameters, but our study BAI score was correlated with TBUT in patients with anxiety while BDI was correlated with TBUT, TMD, TMH and TMA in patients with depression. Taken together, these demonstrate the effects of these diseases itself on ocular surface without any medication. In fact, all previous studies were designed with the patients who were already on psychiatric medication, except for one. [15]

One of the limitations of this study was the small sample size per group and the status for DED and psychiatric problems may vary by day in each participant. Next, we were unable to follow patients for dry eye, after they were started on psychiatric medications, therefore we do not know the effects of these medications.

## Conclusions

In conclusion, we found in our study a strong association between DED and newly diagnosed depression and anxiety. In addition, there were correlations between inventory scores and DED test values. We suggest that ophthalmological examination may be required before starting the patient on psychiatric medication.
